# Draft Genome Sequence of *Pseudomonas* sp. LAB-08 Isolated from Trichloroethene-Contaminated Aquifer Soil

**DOI:** 10.1128/genomeA.00948-16

**Published:** 2016-09-22

**Authors:** Kenshi Suzuki, Fatma A. A. Aziz, Yuma Inuzuka, Yosuke Tashiro, Hiroyuki Futamata

**Affiliations:** a Graduate School of Science and Technology, Shizuoka University, Hamamatsu, Hamamatsu, Japan; b Laboratory of Food Crops, Institute of Tropical Agriculture, Universiti Putra Malaysia, Serdang, Malaysia; c Department of Applied Chemistry and Biochemical Engineering, Graduate School of Engineering, Shizuoka University, Hamamatsu, Japan; d Research Institute of Green Science and Technology, Shizuoka University, Suruga-ku, Shizuoka, Shizuoka, Japan

## Abstract

*Pseudomonas* sp. LAB-08 was isolated from a phenol-fed bioreactor constructed with contaminated aquifer soil as the inoculum. Strain LAB-08 utilized phenol as a sole carbon and energy source. Here, we report the genome sequence and annotation of *Pseudomonas* sp. LAB-08.

## GENOME ANNOUNCEMENT

Trichloroethene (TCE) is used for dry-cleaning and semiconductor manufacturing and its contamination of the subsurface environment is a serious problem for drinking-water sources. TCE is co-metabolically degraded by aliphatic, aromatic, and hydrocarbon-degrading bacteria (1, 2). In our previous work, a positive correlation between affinities for phenol and TCE was found (3). Thus, high-affinity (low *K*_S_ value) TCE-degrading bacteria were enriched in a phenol-fed chemostat (4–6). Here we present the genome sequence of *Pseudomonas* sp. LAB-08 which was isolated from such a chemostat to get insight into the metabolism of phenol.

The genome of strain LAB-08 was sequenced by whole-genome 400-bp shotgun and 300-bp paired-end strategies using 454 GS FLX Titanium (7) and gene walking methods. The 454 GS FLX Titanium data consisted of 700,462 reads and covering 212,407,357 bp. The Newbler GS De Novo Assembler (version 2.5) (7) was used for assembly, resulting in a scaffold (including 16,831 N). The genome was annotated by Rapid Annotation using Subsystem Technology (RAST) (8) and Microbial Genome Annotation Pipeline (MiGAP) (9). The draft genome sequence of strain LAB-08 has a total length of 6,733,957 bp, with an estimated G+C content of 59.2%. The annotated genome includes 6,063 coding sequences (CDSs), seven 5S-16S-23S rRNA clusters, and 73 tRNAs.

During phenol degradation in bacteria, phenol is converted to catechol by phenol hydroxylase (10). Two types of phenol hydroxylase are known, single and multicomponent enzymes (10); multicomponent type hydroxylases are considered predominant in the environment (10). The genome of strain LAB-08 contained one multicomponent phenol hydroxylase. Catechol is converted by either catechol 2,3-dioxygenase (C23O) or catechol 1,2-dioxygenase (C12O), and both products are then converted to acetyl-CoA via *meta*- or *ortho*-cleavage, respectively (11). There were a gene encoding C12O and also the downstream genes of the *ortho*-pathway in the genome of strain LAB-08 but no evidence of C23O. Instead, biphenyl 2,3-dioxygenase, which converts biphenyl-2,3-diol to 2-hydroxy-6-oxo-6-phenylhexa-2, 4-dienoate, and also converts catechol to 2-hydroxymuconic semialdehyde (12) was found. These results suggested that strain LAB-08 has two possible phenol-metabolic pathways and are presumably differentially regulated.

### Accession number(s).

The draft genome sequence of *Pseudomonas* sp. LAB-08 has been deposited at DDBL/GenBank under the accession no. AP017423.
